# Functional Trait-Based Screening of Zn-Pb Tolerant Wild Plant Species at an Abandoned Mine Site in Gard (France) for Rehabilitation of Mediterranean Metal-Contaminated Soils

**DOI:** 10.3390/ijerph17155506

**Published:** 2020-07-30

**Authors:** Isabelle Laffont-Schwob, Jacques Rabier, Véronique Masotti, Hélène Folzer, Lorène Tosini, Laurent Vassalo, Marie-Dominique Salducci, Pascale Prudent

**Affiliations:** 1 Aix Marseille University, IRD, LPED, IRD UMR 151, 13331 Marseille, France; lorene.tosini@univ-amu.fr; 2 Aix Marseille University, Avignon Université, CNRS, IRD, IMBE UMR 7263, 13331 Marseille, France; rabier.jacques3@gmail.com (J.R.); veronique.masotti@imbe.fr (V.M.); helene.folzer@imbe.fr (H.F.); marie-dominique.salducci@imbe.fr (M.-D.S.); 3 Aix Marseille University, CNRS, LCE, UMR 7376, 13331 Marseille, France; Laurent.Vassalo@univ-amu.fr (L.V.); pascale.prudent@univ-amu.fr (P.P.)

**Keywords:** passive ecological restoration, native plant selection, phytoremediation strategy, metal tolerance

## Abstract

The selection of plant species at mine sites is mostly based on metal content in plant parts. Recent works have proposed referring to certain ecological aspects. However, plant traits for plant metal-tolerance still need to be accurately assessed in the field. An abandoned Zn-Pb mine site in Gard (France) offered the opportunity to test a set of ecological criteria. The diversity of micro-habitats was first recorded through floristic relevés and selected categorical and measured plant traits were compared for plant species selection. The floristic composition of the study site consisted in 61 plant species from 31 plant families. This approach enabled us to focus on seven wild plant species naturally growing at the mining site. Their ability to form root symbioses was then observed with a view to phytostabilization management. Four species were considered for phytoextraction: *Noccaea caerulescens* (J. et C. Presl) FK Meyer, *Biscutella laevigata* L., *Armeria arenaria* (Pers.) Schult. and *Plantago lanceolata* L. The metal content of their aerial and root parts was then determined and compared with that of soil samples collected at the same site. This general approach may lead to the development of a knowledge base for assessment of the ecological restoration trajectory of the site and can help in plant selection for remediation of other metal-rich soils in the Mediterranean area based not only on metal removal but on ecological restoration principles.

## 1. Introduction

Heavy metal contaminated soils raise major environmental and human health issues, which may be partially solved by phytoremediation technologies. This cost-effective plant-based approach to remediation takes advantage of the remarkable ability of plants to concentrate trace elements from the environment and to metabolize a large number of molecules in their tissues to reduce their toxicity [1,2]. Consequently, on-site management of heavy metal contaminated soils can be achieved either by using metal hyperaccumulators that are plant species accumulating exceptionally large amounts of heavy metals in their tissues [3] or by using a biocontainment method such as phytostabilization [4]. 

In most of the cases, plant selection is assessed for commercial phytoremediation (preferentially phytoextraction but not excluding phytostabilization) and is designed on agronomy principles using crops and amendments [5,6,7] and far less on the basis of ecological concepts such as trait-based selection [8,9,10].

Initially attracting attention for their potential use in phytomining, hyperaccumulators have been increasingly of interest from an evolutionary or ecological perspective. Studies on hyperaccumulators and more generally on metallophytes have led to a taxonomic approach with a focus on botanical families hosting numerous metal-tolerant species such as Brassicaceae, Poaceae, Fabaceae and Asteraceae in Mediterranean areas. This first selection grid is of interest but remains at a preliminary stage and is not always accurate (i.e., Plantaginaceae). Moreover, facultative hyperaccumulators are dominant in metalliferous soils, and for the purpose of the accurate selection of plant species for metalliferous soil revegetation, it is worthwhile to distinguish species-wide versus population-specific metal tolerance following Pollard et al. [3]. Plant functional traits have been considered as a promising tool for the selection of plants for the purpose of ecological restoration [10]. Life cycle is one of the main traits to be considered for phytostabilization, perennial species being preferred. Most of those recent studies dealt with above-ground-traits and very few with below-ground-traits, which are less easy to access. However, the very first interaction between plant species and trace metals and metalloids (TMM)-rich soil is focused at the root and rhizosphere level. Deep root system and root symbioses are of great importance in the mechanisms of plant tolerance to heavy metals [11,12]. The analysis of the spontaneous processes of plant colonization of abandoned mine sites may contribute to developing better key selection criteria for suitable plant species for on-site TMM management. Moreover, plant ecotypes in the mining sites are tolerant to mixed toxic metals in the soil solution and not only of a single metal such as Zn. For example, it has been recently demonstrated that *Silene vulgaris* ecotypes from calamine soils were fully adapted to heavy metal mixture (Zn, Pb and Cd) compared to ecotypes from both serpentine and non-metallicolous soils [13]. These adaptations depend on their capacity for reactive oxygen species scavenging and TMM compartmentalization in plant parts to avoid their toxicity [2,6]. Selecting ecotypes from a Zn-Pb mining site may be of interest for rehabilitation of multi-metal-contaminated soils.

Our hypothesis is that the flora spontaneously colonizing an abandoned mine site shares common above-ground and below-ground plant traits, i.e., Raunkiær life form, Grime strategy, root system type and, mycorrhizal status. We also considered botanical family as a functional trait, as phylogenetic relatedness is a strong driver for associations with fungal rhizosphere communities [14]. These common features may help in plant species selection for mine rehabilitation. For that purpose, a Zn-Pb-rich abandoned mine site without any previous rehabilitation implemented was selected. The diversity of micro-habitats was recorded through floristic relevés and the selected traits and plant characteristics were compared for plant species selection. A second hypothesis is that phytostabilization and phytoextraction processes may occur concomitantly in abandoned mine sites due to the diversity of plant adaptations in the plant community. This will be discussed through the literature data on plant species known for their hyperaccumulation, phytoaccumulation or phytostabilization abilities.

Using a plant selection grid, seven plant species were selected to study their ability to form root symbioses for the purpose of phytostabilization management. Four plant species were also considered for phytoextraction. The metal content of their aerial and root parts was then determined and compared with that of soil samples collected at the same site. This general approach has led to the development of a knowledge base for the assessment of the ecological restoration trajectory of the site and can help in plant selection for on-site remediation of other metal-rich soil in the Mediterranean area.

## 2. Materials and Methods

### 2.1. Site Location

**Figure 1:**
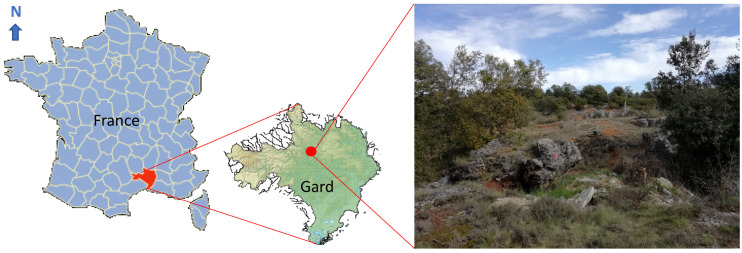
Site location in Gard (France) nearby Rousson and a view of the spontaneous vegetation at the abandoned mine site (March 2020).

The study site is located in the town of Rousson in Gard (Figure 1), and more specifically at a place called La Gardie. It is an abandoned mine site which was used for the extraction of zinc. Established in 1876, the Rousson concession (310 ha) included the sites of Landas, Font de Rouve, and Croix de Fauvie (study site). These deposits, located in interlocking limestones, were superficial deposits made up of pieces of shale in ferruginous soil which contained 20 to 40% of zinc. The ore was processed in calamine furnaces. At the time when this small surface mining operation was closed down in 1910, it employed 8 miners and 5 laborers. Today, there are only a few excavations left that spontaneous vegetation colonization is gradually filling up. These red soils, containing wastes from the mine rich in metals, are potentially colonized by a specific flora adapted to metal-rich soils.

### 2.2. Soil Sampling and Metal Analyses

Samples of soil at the mine site were collected randomly from the top 20 cm after litter removal, then sieved to 2 mm and pooled in three composite samples for metal analysis (Cr, Cu, Fe, Mn, Ni, Pb, Zn). In the laboratory, soil composite aliquots were air-dried at room temperature and then ground to pass through a 0.2 mm titanium sieve (RETSCH zm 1000 with tungsten blades) before analyses. Trace and major metal element concentrations were determined on 3 analytical replicates for each of the three soil composite samples. Soils were mineralized in a microwave mineralizer (Milestone Start D) using *aqua regia* (1/3 HNO_3_ + 2/3 HCl). The mineralization products were filtered with a 0.45 μm mesh and the metal concentrations were determined by ICP-AES (inductively coupled plasma atomic emission spectroscopy, Spectra 2000, Jobin Yvon Horiba group, Longjumeau, France). Quality controls and accuracy were checked using standard soil reference materials (CRM049–050, from RTC-USA) with accuracies within 100 ± 10%.

### 2.3. Experimental Approach for Plant Selection

**Figure 2:**
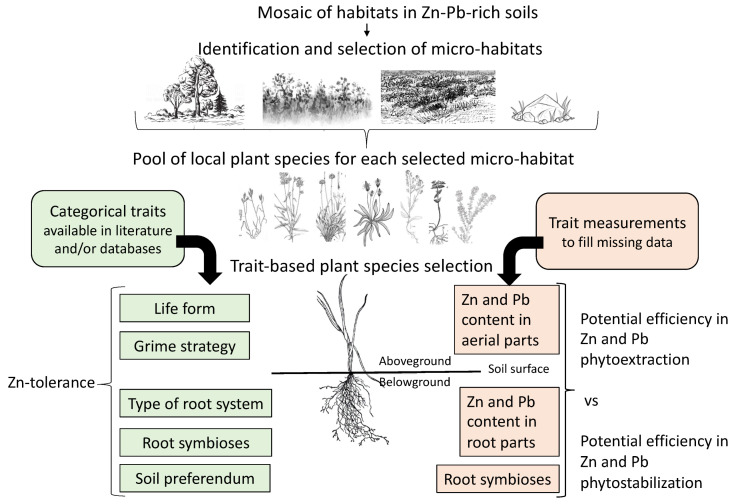
Trait-based grid for local plant selection for phytoremediation of Zn-Pb-rich soils from an ecological restoration perspective.

The main aim is to assess the efficiency of a trait-based approach for phytoremediation plant selection in the field. To achieve this aim, a hierarchical method was designed based on analysis of literature data with experimental measurements to fill the gaps in knowledge, as described in Figure 2.

### 2.4. Floristic Analysis and Habitat Inventory

**Figure 3:**
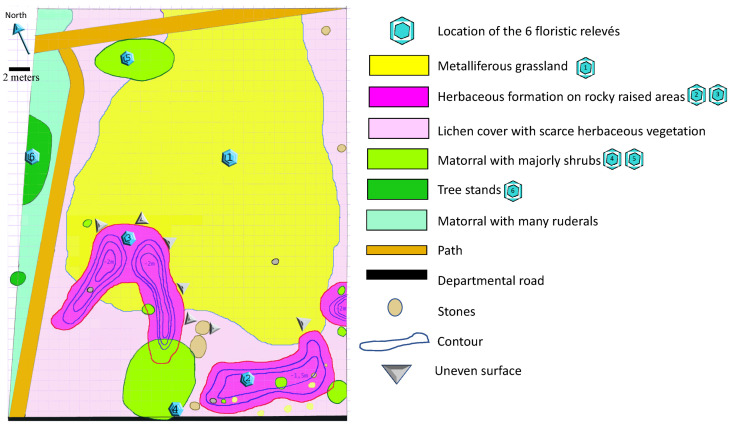
Cartography of the mosaic of micro-habitats at an abandoned mine site at La Gardie. Icons with number indicate the number of the relevé and its location in the field (bar = 2 m).

The study was done during spring in a defined area of 30 m^2^ representative of an excavation in the abandoned mining area with spontaneous vegetation colonization. Vertical (herbaceous, shrub and tree strata) and horizontal (global plant cover) structures of plant communities were used to distinguish the different micro-habitats (Figure 1, Figure 2 and Figure 3). Within each type of micro-habitat, representative areas characterized by the homogeneity of vegetation were selected for floristic analysis. The micro-habitats were classified based on plant species composition (presence/absence). Finally, a global analysis on 6 relevés was done on the four selected micro-habitats in terms of plant species occurrence.

### 2.5. Bibliographical Functional Traits

Functional traits such as type of root, Grime strategy, Raunkiær biological type, and mycorrhizal status were collected from a review of scientific papers for each of the identified plant species in the field. The functional response traits were selected with the aim of better understanding the influence that these plant species may have on the passive phytoremediation processes.

### 2.6. Plant Analyses

#### 2.6.1. Root Symbiosis Assessment

Seven plant species were selected, i.e., *Armeria arenaria* (Pers.) Schult., *Biscutella laevigata* L., *Brachypodium phoenicoides* (L.) Roem. & Schult, *Trifolium pratense* L., *Mibora minima* (L.) Desv., *Noccaea caerulescens* (J. et C. Presl) FK Meyer and *Thymus vulgaris* L. for assessment of their root symbioses in the field. The selection was done after literature review, and only plant species for which there was a lack of knowledge, scarce data or controversial status were chosen. Thin roots were randomly selected in the root system of 3 individuals of each of the selected plant species. The root samples were first rinsed under tap water then with deionized water and stored in alcohol (60%, *v/v*) at room temperature until proceeding. The occurrence of symbiont colonization was estimated by visual observation of fungal colonization after clearing roots in 10% KOH and staining with lactophenol blue solution, according to Phillips and Hayman [15].

#### 2.6.2. Metal Content in Below-Ground and Above-Ground Plant Parts

Four plant species were selected for metal analysis, i.e., *N. caerulescens*, *B. laevigata*, *A. arenaria* and *Plantago lanceolata* L. Dried plant samples (root and aerial parts, separately) were ground to pass a 0.2 mm mesh titanium sieve, and three aliquots were analyzed per sample. About 0.5 g of dry matter was digested with the microwave digestion system Milestone start D with a HNO_3_-HCl mixture (volume proportion ratio 2/1). After filtration (0.45 µm), acid digests were analyzed for metal content by ICP-AES (JY 2000 Jobin Yvon Horiba group, Longjumeau, France). Standard plant reference material (DC 73,349) from the China National Analysis Centre for Iron and Steel (NCS), was analyzed as a part of the quality control protocol (accuracies within 100 ± 10%).

### 2.7. Statistical Analysis

Statistical analyses were performed for all data using JMP 12 statistical software (SAS Institute, Cary, NC, USA) using the non-parametric Kruskal-Wallis test because data did not follow a normal distribution pattern. The nonparametric pairwise multiple- comparison Dunn’s test was used when the null hypothesis was rejected with the Kruskal–Wallis test.

## 3. Results and Discussion

### 3.1. Soil Contamination

Soils contained high concentrations of zinc (ca.11.7%) and lead (ca.1.7%) as expected in this type of abandoned mining area (Table 1). Moreover, elevated concentrations in Fe, Mn, and Cr were also detected. Zn and Pb soil concentrations were higher than those previously reported in mine tailings in Spain, i.e., highest concentrations ranging between ca. 2360, 7000 and 11,600 mg/kg of Zn in the Alcudia Valley, San José heaps and El Lirio tailing, respectively and ca. 7000 and 10,000 mg/kg of Pb in Belleza tailing and the Alcudia Valley, respectively [16,17,18]. However higher Zn soil content was detected at Mánforas with 135,080 mg/kg [17]. In Portugal, up to 9330 mg/kg of Pb were observed in abandoned Pb mines [19]. Compared to mine sites in France, at the Les Malines and Les Avinières near the present study site, the authors detected lower concentrations in Zn and greater in Pb, i.e., 59,040 mg/kg of Zn and 62,051 mg/kg of Pb [20]. In Poland, in reclaimed soils of Zn-Pb mine wastes, up to 7.57% of Zn and 0.46% of Pb were found [21]. Such heavy metal-rich soils may not be used for agriculture purposes, and the spontaneous plant colonization that occurred after operation ceased was a great opportunity to reduce the transfer of elements into the food web and to reduce the environmental and human exposure.

### 3.2. Diversity of Habitats and Plant Species

A total of 4 micro-habitats were identified after preliminary cartography of the 30 m^2^ study site, consisting of (i) grassland colonizing metal-rich soil, (ii) herbaceous colonization of rocky soils, (iii) matorral with shrub dominating and (iv) woody vegetation (Figure 3). Two supplementary micro-habitats were removed from the inventory. The first concerned an area in which the major part was covered by lichens belonging to 2 species, i.e., *Cladonia rengiformis* and *Cladonia foliacea* s.l., beyond the scope of the study. A second area near the road was mostly constituted of ruderals due to the modified substrate of the road foundation.

The floristic composition of the study site consisted in 61 plant species growing wild on these 4 micro-habitat types and identified during the spring period (Table 2). It included 31 botanical families at the period of the relevés. In terms of species diversity, the Poaceae family was the most frequent (8 species), then the Brassicaceae and Rosaceae with 5 species each. Asteraceae occurred with 3 species, followed by Fabaceae, Caryophyllaceae, Rubiaceae and Ranunculaceae (Table 2).

In terms of estimated plant cover, the dominant plant species were Brassicaceae with mainly two species (*B. laevigata* and *N. caerulescens*), Poaceae (*B. phoenicoides, M. minima* and *Festuca ovina* L.), Plumbaginaceae (*A. arenaria*), Fabaceae (*T. pratense*). and Lamiaceae (*T. vulgaris*).

### 3.3. Analysis of Plant Traits Linked with TMM Tolerance

From the literature, different plant traits commonly used in different floristic analyses of spontaneous colonization of mining sites were assessed for all the 61 identified plant species (Table A1). Those categorical traits may help us to understand the distribution pattern of plant species along environmental gradients [22]. However, their local variability needs to be discussed, and continuous traits need to be locally assessed to test the robustness of this approach.

#### 3.3.1. Categorical Trait Analysis of the 61 Identified Plant Species

Considering below-ground parts, 61% of the plant species were characterized by fibrous root systems, 26% by rhizome or tuberous root system and only 13% by slender roots. A hypothesis has been formulated by Sardans and Peñuelas [23] and confirmed by Pierret et al. [24] considering deep roots as able to access water and nutrients in deep layers of soil and/or fractured bedrock, which are unavailable to surface roots. This mechanism will potentially help to maintain higher moisture levels in the upper soil layers and could be a factor explaining the high plant diversity in these dry habitats, despite the water stress due to both Mediterranean conditions and high salt concentration in the water linked to the metalliferous soils.

The mycorrhizal status of most of these 61 plant species has already been described [25,26,27]. Out of all the plant species, 67% were associated with arbuscular endomycorrhizal fungi, ca. 5% with ectomycorrhizal fungi and 8% were reputed to share no mycorrhizal interactions. For ca. 20% of the identified plant species, no information regarding their mycorrhizal status has been reported to the best of our knowledge.

Concerning the preferential type of soil, 44% of the plant species were adapted to dry and rocky soils, ca. 20% specific to calcareous soils, ca. 20% preferred sandy or well-drained soils and 16% were mostly found in disturbed or cultivated soils.

**Figure 4:**
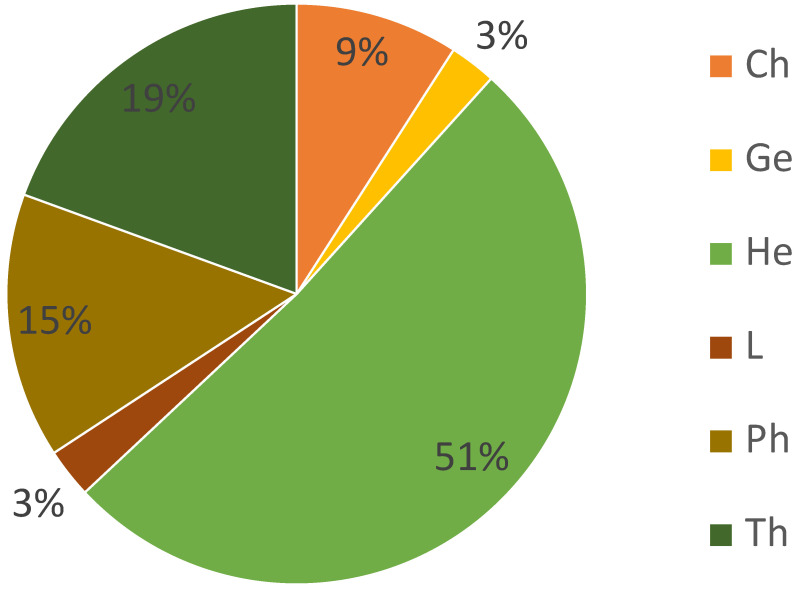
Average percentage of each life form in the studied mosaic of habitats at the former mine site. Ph: Phanerophytes; He: Hemicryptophytes; Ch: Chamaephytes; L: Liana; Ge: Geophytes; Th: Therophytes.

The dominant life form (Figure 4) were hemicryptophytes with an average percentage of 51%, then therophytes (19%) and phanerophytes (15%). Chamaephytes only represented an average of 9%. Hemicryptophytes and therophytes were found in all of the 6 relevés although the occurrence of phanerophytes varied with none of this life form in the center of the area (open dry grasslands) and 31% in the tree stand (Table 2 and Table A1). Geophytes and lianas were only identified in 2 and 3 out of 6 relevés, respectively.

**Figure 5:**
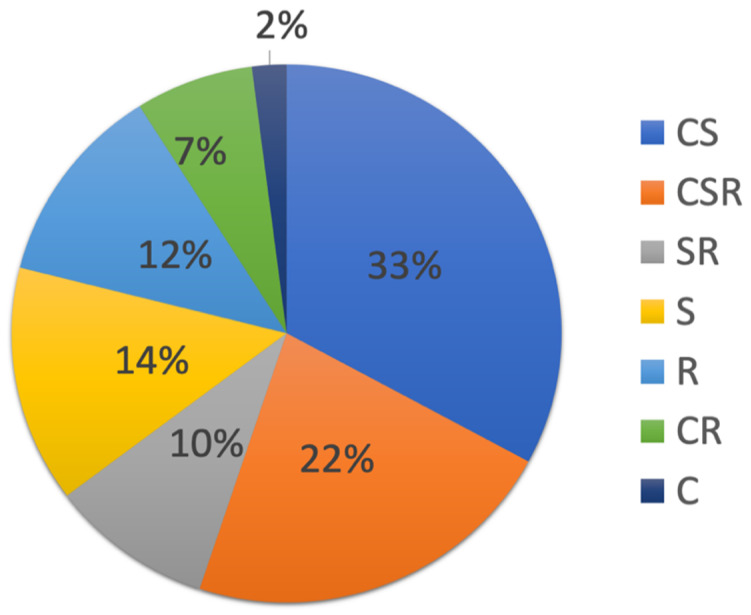
Average percentage of each Grime strategy in the studied mosaic of habitats at the former mine site. CS: competitive, stress tolerant; CSR: competitive, stress tolerant, ruderal; SR: stress tolerant, ruderal; S: stress tolerant; R: ruderal; CR: competitive ruderal; C: competitive.

Concerning plant strategy, 33% of species were considered as CS, i.e., competitive/stress tolerant, and 22% CSR, i.e., competitive, stress tolerant, ruderal (Figure 5).

#### 3.3.2. Analysis of Plant Traits by TMM Tolerance Strategy

**Figure 6:**
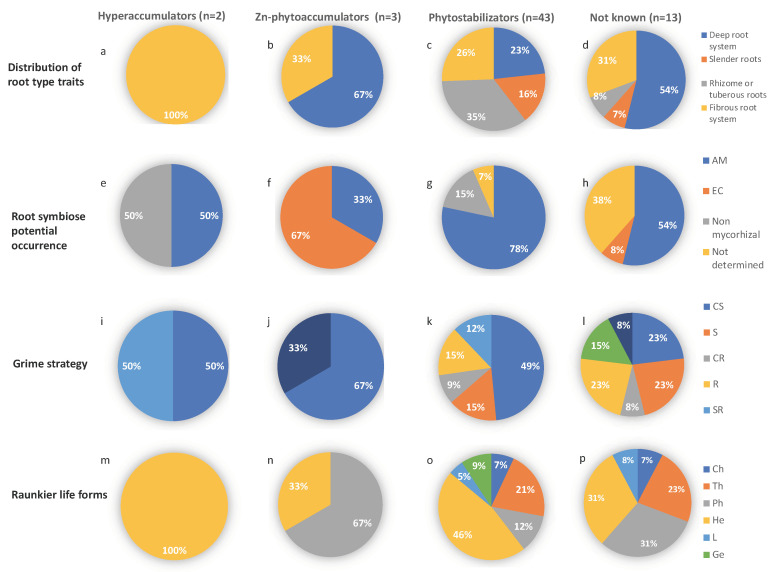
Distribution pattern of root type traits (**a**–**d**), root symbiosis potential occurrence (**e**–**h**), Grime strategy (**i**–**l**) and Raunkiær life forms (**m**–**p**) by TMM tolerance strategy, i.e., hyperaccumulators (*N. caerulescens* (Zn/Cd/Pb- hyperaccumulator) and *B. leavigata* (Tl/Pb/Cd- hyperaccumulator)), Zn-phytoaccumulators (*Plantago lanceolata*, *Quercus ilex* and, *Pinus sylvestris*), phytostabilizators or not known in the literature (see Table A1).

The identified plant species were grouped in hyperaccumulators, phytoaccumulators, phytostabilizators or not known in the literature. Using this grid of comparison, the above-cited traits were analyzed (Figure 6). Even if the number of species considered as hyperaccumulators (2) or as phytoaccumulators (3) is limited in the study field, some traits are typical of the plant strategy. The dominant root system was fibrous for the two hyperaccumulators (Figure 6a) and deep root system for phytoaccumulators (Figure 6b). No specific root type could be defined for phytostabilizators (Figure 6c). For phytostabilization strategy (43), identified plant species in the study site had mainly rhizomes or tuberous roots (35%, Figure 6c), ca. all of them were known to be endomycorrhized (78%, Figure 6g), the dominant Grime strategy was CS (49%, Figure 6k), and the main life form was hemicryptophyte (46%, Figure 6o). These results are in accordance with many previous studies demonstrating that only a few spontaneous plant species colonizing mine sites may favor heavy metal translocation in the aerial parts and may be useful for phytoextraction. Most of the metal tolerant plant species may accumulate heavy metals in their roots and limit their transfer to the aerial parts, being potentially useful in phytostabilization. Without any human intervention by plant harvesting, the dominant natural process is therefore phytostabilization. Hemicryptophyte life form was strongly present in the three groups (Figure 6m–o). Hemicryptophytes such as biannuals or with thin root systems may limit the phytostabilization potential. On the other hand, the non-perennial aerial parts of hemicryptophytes may also limit the phytoextraction ability. The traits that most discriminated hyperaccumulators from both phytoaccumulators and phytostabilizators were the ability to form arbuscular mycorrhizal (AM) associations (50% non-mycorrhizal for the first and 33% and 78% AM for the other two; Figure 6e, f and g). It is noteworthy that ectomycorrhizal (EC) type was dominant (67%) in phytoaccumulators. It is congruent with the 67% of phanerophyte type. *Quercus ilex* and *Pinus sylvestris* are known to be predominantly associated with ectomycorrhizal fungi [26].

#### 3.3.3. Measured Trait Analysis on Selected Plant Species

Mycorrhizal status of 7 selected plant species: an efficient tool for plant species selection for phytostabilization strategy?

The mycorrhizal status of *A. arenaria*, *B. laevigata*, *B. phoenicoides*, *T. pratense*, *M. minima*, *N. caerulescens* and *T. vulgaris* was assessed.

**Figure 7:**
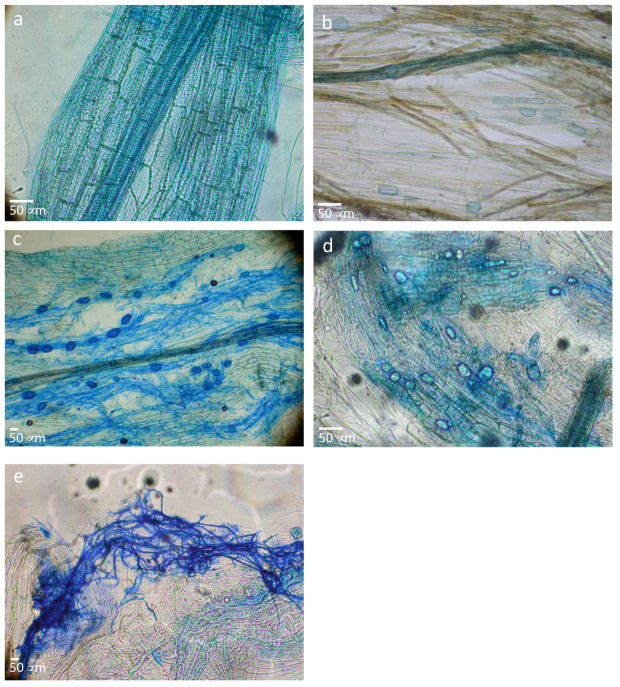
Microphotographs of roots stained for mycorrhizal association observation: root parts from *N. caerulescens* (**a**), *A. arenaria* (**b**), *T. pratense* (**c**), *B. phoenicoides* (**d**) and, *T. vulgaris* (**e**). AM mycelium and vesicles appear in dark blue. Scale bars are directly on the microphotographs.

*N. caerulescens* (Figure 7a) and *B. laevigata* (not shown) were not mycorrhized at this site. However, there is no consensus regarding the mycorrhizal status of *B. laevigata* and *N. caerulescens*, long considered not to be mycorrhized; some authors detected AM associations with both species [26,27,28].

*N. caerulescens* (Figure 7a) and *B. laevigata* (not shown) were not mycorrhized at this site. However, there is no consensus regarding the mycorrhizal status of *B. laevigata* and *N. caerulescens*, long considered not to be mycorrhized; some authors detected AM associations with both species [26,27,28].

A total of 20% of root length colonization by AM fungi was detected for *A. arenaria* (Figure 7b) although no AM colonization was revealed for *M. minima* (not shown). No previous data regarding the mycorrhizal status of *A. arenaria* and *M. minima* have been published. To the best of our knowledge, this is the first report of AM association with *A. arenaria.* With regard to *M. minima*, the smallest Poaceae occurring in France, it is also the first report concerning its mycorrhizal status. Its very short life cycle (few months) could be linked to a lack of AM association.

Wang and Qiu [26] and Pawlowska et al. [27] gave discordant results for *T. pratense.* At the present site, AM mycelia were abundant, and many spores were detected in roots of *T. pratense* with an overall colonization ca. 80% of root length (Figure 7c).

*B. phoenicoides* mycorrhizal status has previously been analyzed [29]. However, data are scarce. In our study, this species appeared to be endomycorrhized (Figure 7d). *T. vulgaris* was the only Lamiaceae identified at our study site and since Lamiaceae are usually good candidates for phytostabilization, we endeavored to confirm its mycorrhizal status. This perennial developed AM association and a dense web of mycelia was observed with many vesicles (Figure 7e).

The occurrence of AM symbioses is a strong advantage for phytostabilization but not specific to this type of process. Mycorrhizal associations may favor TMM extraction by enhancing metal mobilization [30]. Therefore, this trait alone would not be a good criterium for plant species selection for the purpose of phytostabilization strategy.

Plant colonization at mining sites may be favored by AM fungi, the latter, which may lower metal toxicity and improve nutrient availability for plants. However, in a Zn-, Cd-, Pb-, and Cu-polluted field study, no evidence for an effect of AM symbioses has been found on plant metal uptake [31]. Therefore, with regard to a phytoextraction strategy, the authors suggest not channeling efforts exclusively towards reinforcing AM symbiosis. In a recent review paper dealing with AM associations at mining sites, it was observed that more than 80% of the plant species from metallic mines were endomycorrhized suggesting adaptive strategies in coevolved fungal strains and plant species [32]. Rehabilitation of metal-rich soils without metal removal may be achieved by selecting plant species with their co-evolved mycorrhizal symbionts.

#### 3.3.4. Metal Content in 4 Plant Species for Phytoextraction Strategy

In agreement with the literature, *N. caerulescens* was the best accumulating species out of the 4 selected for zinc and lead (Table 3), and it is defined as a facultative Zn-hyperaccumulator [3]. This species also accumulated high levels of Fe in its aerial parts. Furthermore, *B. laevigata* and *A. arenaria* seemed to be suitable as Zn-phytoaccumulators with more than 1000 mg/kg (dry matter, DM) of zinc accumulated in their aerial parts. Those species have already been identified as valuable temperate zone phytoaccumulators of Zn and Cd. *P. lanceolata*, in a lesser way, appeared as a good Zn phytoaccumulators. Zn content up to 430 mg/kg in shoots of *P. lanceolata* was previously reported in a mining area of Southern Poland [33] although 946 mg/kg were detected in *P. lanceolata* aerial parts in the present study. All these four plant species are also potentially good candidates in Mediterranean areas. Although average soil Pb concentration was high (Table 1), this element was moderately accumulated in the aerial parts of *N. caerulescens* (ca. 496 mg/kg) and ranged from 48 to 75 mg/kg in the aerial parts of the three other plant species.

Apart from Fe, Zn and Pb were the studied elements most translocated to the aerial parts of the four plant species. However, Pb, as a non-essential element, may be transferred to aerial parts via transporters of other elements essential to plants. This type of elemental competition between nutrients and toxic metals is most of the time antagonistic. However, the present results show concomitant translocation of Zn and Pb. Our results demonstrated similar results for *N. caerulescens*, *A. arenaria*, *B. laevigata* and *P. lanceolata*. *N. caerulescens* is sometimes considered as a Zn-hyperaccumulator and Pb co-accumulator [34]. A previous work on *N. caerulescens* also shows a negative correlation between Zn content in shoot parts and Ca and Mg concentrations in shoot parts due to competition between these different cations [13]. Synergistic and antagonistic interactions for element absorption in plants is a challenging field of research and there is still a need for more knowledge. However, these preliminary results showed the interest of these plant species in Zn and Pb-rich soils.

#### 3.3.5. Spontaneous Plant Colonization at Abandoned Zn-Pb Mine Sites: A Matter of Geographical Situation or Plant Traits?

Out of the 61 plant species identified at the abandoned mine site, 47 were already known to grow in Zn-rich soils. Among the 14 other plant species, 4 are phanerophytes and may have slowly colonized the site. Five have a reduced biomass and a low cover potential. Four plant species are typical of Mediterranean environments, and the others have a wider range of distribution. Some previous studies, notably at an abandoned Zn-mine site and in metalliferous soils in Greece, Poland, Spain, Italy, Portugal and France, have detailed the floristic composition and some of their plant traits [17,19,20,27,35,36,37] enabling a comparison of occurrence of plant species even though these studies were conducted under varying biogeographical conditions. Out of the seven relevés from the cited literature, *Rubus ulmifolius* Schott and *P. lanceolata* were the most frequently identified species on these sites. *B. leavigata, D. glomerata* and *F. ovina* occurred in 4 out of the 7 relevés in France, Poland and/or Portugal. *N. caerulescens* only occurred in the 2 relevés from France. Hemicryptophytes were dominant in most of the studies [20,38] including in the present study. No systematic selection could be made solely on the basis of plant traits, but common features are shared by the spontaneous vegetation of the European and Mediterranean abandoned Zn-mine sites that it might be useful to identify with a view to the ecological restoration of Zn-rich soils.

It is worth noticing that, in many studies, the occurrence of certain exotic phanerophytes was recurrent, such as *Eucalyptus globulus* [19], but these species are not to be encouraged in ecological restoration processes; it would be better to focus on native plant species.

On the basis of all the information collected through the diverse relevés, it appeared that some ubiquitous Zn-tolerant plant species in Mediterranean and European areas are potentially good candidates for the first stages of ecological restoration of Zn-Pb-rich soils. Previous restoration programs in Poland have used *D. glomerata* and *T. pratense,* also identified at the present study site. However, this was done with selected cultivars and not seedlings of wild origin [39]. This previous integrative study has suggested various potential lines of research including endomycorrhizal inoculation before transplantation with selected fungal strains and also selection of xerothermic plant species to cope with water stress. Their feedback shows how important the development of rhizosphere microorganisms consortia is for successful site restoration [39]. AM colonization was also a dominant trait as previously reported [27]. However, local plant associations should be favored and the geographical situation needs to be taken into account. Moreover, more than the below-ground parts of the plants, rhizosphere micro-organisms are potentially the key factor for plant colonization in these metal-rich soils following the mycorrhizal fungal diversity–ecosystem function concept of Hazard and Johnson [40]. Plant functioning (nutrition, stress-tolerance, etc.) is intrinsically dependent on associated micro-organisms [41].

## 4. Conclusions

This work enabled the implementation of a plant database consisting of above-ground and below-ground plant traits of plant species able to grow on Zn-Pb rich soils. This preliminary work may be a useful tool for plant selection in rehabilitation programs for Zn-Pb-rich soils. It also highlighted the need for more information regarding below-ground traits and rhizosphere microbial consortia.

The trait-based analysis provided a basis for drawing a general picture of plant communities in a Mediterranean abandoned Zn-Pb mine site. Below-ground traits appeared as important features for phytostabilization. Ectomycorrhizae were dominant in the Zn-phytoaccumulators species and AM, in the phytostabilizators. Together, these plant strategies may favor fungal interaction diversity and enhance the sustainability of the plant-fungal communities.

The four plant species selected for phytoextraction, i.e., *N. caerulescens*, *B. laevigata*, *A. arenaria* and *P. lanceolata* showed interesting Pb and Zn accumulation capacities in their aerial parts. However, these plant species are of reduced biomass and a phytoextraction process with those species would not be efficient. Those plant species are however interesting in the early plant succession stage after mines are abandoned. Among the identified potential phytoaccumulators, *Quercus ilex* and *Pinus sylvestris*, both phanerophytes, are long-term colonizers and persistent plant species. However, management strategies based on tree plantation would create more litter with reduced undergrowth. With long-term Zn potential accumulation in the aerial parts, there is a need for further knowledge regarding the risk of Zn transfer into the food web.

Plant communities at the mine site mostly favored a passive phytostabilization that is maintained over time by seasonal turnover of therophytes and persistence of belowground parts of hemicryptophytes and geophytes and both belowground and aboveground parts of chamaephytes and phanerophytes. Only few plant species were potentially able to accumulate Zn and Pb in the aerial parts. Moreover, a phytoextraction process requires human intervention by removal of metal-rich aerial parts. Consequently, even if both strategies occurred in these plant communities, the overall trend is the immobilization of heavy metals in the soils and root systems.

## Figures and Tables

**Table 1:** Average soil metal content (mg/kg dry weight) at La Gardie mine site.

Element (mg/kg)						
Cr	Cu	Fe	Mn	Ni	Pb	Zn
103 ± 38	39 ± 17	173,377 ± 58,177	3000 ± 936	89 ± 30	17,230 ± 6804	117,321 ± 33,770

Values are means of triplicates.

**Table 2:** Floristic list established during springtime and occurrence of each plant species in the 6 relevés out of the 4 selected micro-habitats encountered at the former mine site. * (1): Metalliferous grassland; (2) and (3): Herbaceous formation on rocky raised areas; (4) and (5): Matorral with majorly shrubs; (6): Tree stands and frequency out of the 6 relevés.

Latin Name	Botanical Family	Occurrence in Each Relevé *						Frequency
		(1)	(2)	(3)	(4)	(5)	(6)	-
*Amelanchier ovalis* Medik.	Rosaceae	-	-	-	-	-	X	1/6
*Aphyllantes monspeliensis* L.	Liliaceae	-	X	-	-	X	-	2/6
*Arenaria serpyllifolia* L.	Caryophyllaceae	-	-	X	X	X	X	4/6
*Argyrolobium zanonii* (Turra) P.W.Ball	Fabaceae	-	X	-	X	-	X	3/6
*Armeria arenaria subsp bupleuroides* (Godr. & Gren.) Greuter & Burdet	Plumbaginaceae	X	X	X	X	-	-	4/6
*Asparagus acutifolius* L.	Asparagaceae	-	-	-	X	-	-	1/6
*Asplenium ruta-muraria* L.	Aspleniaceae	-	X	-	-	-	-	1/6
*Biscutella laevigata* L.	Brassicaceae	X	X	X	X	X	X	6/6
*Brachypodium phoenicoides* (L.) Roem. & Schult	Poaceae	-	-	-	-	-	X	1/6
*Brachypodium retusum* (Pers.) P.Beauv.	Poaceae	-	-	-	-	-	X	1/6
*Bromus madritensis* L.	Poaceae	-	-	-	X	-	-	1/6
*Buxus sempervirens* L.	Buxaceae	-	X	X	X	-	X	4/6
*Carex halleriana* Asso	Cyperaceae	-	-	-	-	X	-	1/6
*Centaurea pectinata* L.	Asteraceae	X	X	X	X	-	-	4/6
*Cerastium pumilum* Curtis	Caryophyllaceae	-	X	X	-	-	X	3/6
*Clematis vitalba* L.	Ranunculaceae	-	-	-	-	-	X	1/6
*Clinopodium nepeta* (L.) Kuntze	Lamiaceae	-	-	-	-	X	X	1/6
*Dactylis glomerata* L.	Poaceae	-	-	-	-	X	X	2/6
*Dioscorea communis* (L.) Caddick & Wilkin	Dioscoreaceae	-	-	-	-	-	X	1/6
*Draba verna* L.	Brassicaceae	-	X	X	-	-	-	2/6
*Eryngium campestre* L.	Apiaceae	-	-	-	X	-	-	1/6
*Euphorbia cyparissias* L.	Euphorbiacee	-	X	X	X	-	-	3/6
*Festuca ovina sl* L.	Poaceae	X	X	X	-	X	X	5/6
*Galium aparine* L.	Rubiaceae	-	X	X	X	X	-	4/6
*Galium corrudifolium* Vill.	Rubiaceae	-	-	-	-	X	X	2/6
*Helleborus foetidus* L.	Ranunculaceae	-	-	-	-	-	X	1/6
*Hordeum murinum* L.	Poaceae	-	-	-	X	-	-	1/6
*Hornungia petraea* (L.) ex Rchb.	Brassicaceae	-	X	X	-	-	-	2/6
*Hypericum perforatum* L.	Hypericaceae	-	X	-	-	-	-	1/6
*Juniperus oxycedrus* L.	Cupressaceae	-	-	-	X	-	X	2/6
*Lactuca perennis* L.	Asteraceae	-	-	-	-	-	X	1/6
*Lepidium draba* L.	Brassicaceae	-	-	-	X	-	-	1/6
*Lysimachia arvensis* (L.) U. Manns & Anderb.	Primulaceae	-	-	-	X	X	-	2/6
*Mibora minima* (L.) Desv.	Poaceae	X	X	X	X	-	-	4/6
*Noccaea caerulescens* (J.Presl & C.Presl) F.K.Mey	Brassicaceae	X	X	X	X	X	-	5/6
*Pilosella officinarum* Vaill.	Asteraceae	-	X	X	X	-	-	3/6
*Pinus sylvestris* L.	Pinaceae	-	X	X	-	-	X	3/6
*Pistacia terebinthus* L.	Anacardiaceae	-	-	-	-	-	X	1/6
*Plantago lanceolata* L.	Plantaginaceae	-	-	-	-	-	X	1/6
*Poa annua* L.	Poaceae	-	-	-	X	-	-	1/6
*Poterium sanguisorba* L.	Rosaceae	-	-	-	-	-	X	1/6
*Pyrus spinosa* Forssk.	Rosaceae	-	-	-	-	-	X	1/6
*Quercus* ilex L.	Fagaceae	-	X	X	X	X	X	5/6
*Quercus pubescens* Willd.	Fagaceae	-	-	-	-	X	X	2/6
*Ranunculus bulbosus* L.	Ranunculaceae	-	-	-	-	X	X	2/6
*Reseda lutea* L.	Resedaceae	X	X	X	X	-	-	4/6
*Rosa canina* L.	Rosaceae	-	-	-	-	-	X	1/6
*Rubia peregrina* L.	Rubiaceae	-	-	-	X	-	X	2/6
*Rubus ulmifolius* Schott	Rosaceae	-	-	-	X	-	X	2/6
*Rumex intermedius* D.C.	Polygonaceae	-	X	X	-	-	-	2/6
*Ruscus aculeatus* L.	Asparagaceae	-	-	-	-	X	X	2/6
*Scabiosa atropurpurea* L.	Caprifoliaceae	X	X	X	-	-	-	3/6
*Scrophularia lucida* L.	Scrophulariaceae	-	X	X	-	-	-	2/6
*Sedum acre* L.	Crassulaceae	-	-	-	X	-	-	1/6
*Sedum annuum* L.	Crassulaceae	-	X	X	-	-	-	2/6
*Senecio vulgaris* L.	Asteraceae	-	-	-	X	-	-	1/6
*Silene vulgaris* (Moench) Garcke	Caryophyllaceae	-	-	-	X	-	-	1/6
*Smilax aspera* L.	Smilacaceae	-	X	-	X	-	X	3/6
*Thymus vulgaris* L.	Lamiaceae	X	X	X	X	X	X	6/6
*Trifolium pratense* L.	Fabaceae	-	-	-	-	X	X	2/6
*Ulex parviflorus* Pourr.	Fabaceae	-	X	-	X	-	-	2/6

X: found on the plot.

**Table 3:** Aerial and root part metal content (mg/kg dry weight) at La Gardie mine site.

Element (mg/kg)	Plant Species			
	*A. arenaria*	*B. laevigata*	*N. caerulescens*	*P. lanceolata*
In aerial parts				
Cr	9.6 ± 4.3	10.7 ± 5.3	11.4 ± 4.2	11.4 ± 4.2
Cu	4.6 ± 0.3	3.9 ± 0.5	5.0 ± 1.3	7.3 ± 0.4
Fe	966.3 ± 375.4	325.6 ± 186.2	5780 ± 1878	728.5 ± 167.1
Mn	160.3 ± 99.1	204.9 ± 80.2	105.3 ± 25.0	36.6 ± 4.0
Ni	1.8 ± 0.2 ^ab^	1.8 ± 0.3 ^ab^	3.6 ± 0.8 ^a^	0.5 ± 0.2 ^b^
Pb	74.9 ± 6.8 ^ab^	48.2 ± 6.3 ^b^	496.2 ± 104.6 ^a^	69.2 ± 15.1 ^ab^
Zn	1,916 ± 201 ^ab^	1,775 ± 125 ^ab^	10,664 ± 563 ^a^	946 ± 88 ^b^
In root parts				
Cr	4.3 ± 1.9	2.5 ± 0.8	29.9 ± 9.1	10.1 ± 0.5
Cu	8.6 ± 1.7	6.9 ± 0.1	27.2 ± 6.7	21.3 ± 1.2
Fe	7,719 ± 3,565	5,472 ± 1,009	56,779 ± 24,995	15,210 ± 1,035
Mn	303.0 ± 89.7	223.2 ± 46.3	1,143.2 ± 427.7	405.7 ± 37.9
Ni	5.2 ± 2.5	5.9 ± 2.5	30.6 ± 13.7	7.8 ± 0.6
Pb	1,769 ± 49	1,393 ± 129	5,123 ± 1,879	1,630 ± 124
Zn	10,659 ± 2,763	6,902 ± 547	66,381 ± 24,010	12,217 ± 996

Values are means of triplicates. Means followed by different letters (^a,b, ab^) are significantly different (Dunn test, *p* ≤ 0.05).

## Appendix A

**Table A1:** Floristic list established during springtime from the mosaic of habitats encountered in the former mine site. Traits selected from literature: Root type; Root symbioses: AM: Arbuscular mycorrhizae, EC: Ectomycorrhizae; NM: Non mycorrhizal; Soil preferendum; Life forms [42]: Ph: Phanerophytes; He: Hemicryptophytes; Ch: Chamaephytes; L: Liana; Ge: Geophytes, Th: Therophytes; Grime strategy: CS: Competitive stress tolerant; CSR: Competitive, stress tolerant, ruderal; SR: Stress tolerant, ruderal; S: Stress tolerant; R: Ruderal; CR: Competitve ruderal; C: Competitive; Zn tolerance.

Latin Name	Botanical Family	Root System	Root Symbioses	Soil Preferendum	Life Form	Grime Strategy	Zn Tolerance
*Amelanchier ovalis* Medik.	Rosaceae	Deep root system	AM [43]	Rocky dry soils	Ph	CS	Not known
*Aphyllantes monspeliensis* L.	Liliaceae	Fibrous root system	Not known	Dry soils	He	S	Not known
*Arenaria serpyllifolia* L.	Caryophyllaceae	Thin root system	NM [44,45]	Sandy and rocky soils, calcareous soils	Th	R	Population-specific Zn tolerance [46]
*Argyrolobium zanonii* (Turra) P.W.Ball	Fabaceae	Fibrous root system	AM [47]	Calcareous soils	Ch	S	Not known
*Armeria arenaria* subsp *bupleuroides* (Godr. & Gren.) Greuter & Burdet	Plumbaginaceae	Fibrous root system	Not known	Sandy and rocky soils	He	S	Population-specific Zn tolerance [48]
*Asparagus acutifolius* L.	Asparagaceae	Rhizome	AM [26]	Dry sand arid oils	He	CS	Population-specific Zn tolerance [20]
*Asplenium ruta-muraria* L.	Aspleniaceae	Rhizome	AM + NM [26]	Cliffs, rocks	He	CS	Population-specific Zn tolerance [49]
*Biscutella laevigata* L.	Brassicaceae	Fibrous root system	NM [26]/AM [27,28]	Rocky soils	He	SR	Species-wide Zn tolerance [8], Tl-, Cd-hyperaccumulator [50]
*Brachypodium phoenicoides* (L.) Roem. & Schult	Poaceae	Rhizome	AM [29]	Dry soils	He	CS	Population-specific Zn tolerance [19]
*Brachypodium retusum* (Pers.) P.Beauv.	Poaceae	Rhizome	AM [26]	Dry soils	He	CS	Population-specific Zn tolerance [8]
*Bromus madritensis* L.	Poaceae	Fibrous root system	AM [26]	Sandy soils, cultivated soils	Th	SR	Population-specific Zn tolerance [51]
*Buxus sempervirens* L.	Buxaceae	Deep root system	AM [26]	Dry calcareous soils	Ph	CS	Population-specific Zn tolerance [35]
*Carex halleriana* Asso	Cyperaceae	Rhizome	NM [52]	Dry calcareous soils	He	CSR	Population-specific Zn tolerance [37]
*Centaurea pectinata* L.	Asteraceae	Deep root system	Not known	Rocky soils	He	S	Population-specific Zn tolerance [20]
*Cerastium pumilum* Curtis	Caryophyllaceae	Slender root system	Not known	Sandy soils	Th	SR	Population-specific Zn tolerance [53]
*Clematis vitalba* L.	Ranunculaceae	fibrous root system	AM [26]	calcareous to acidic soils	L	CS	Population-specific Zn tolerance [54]
*Clinopodium nepeta* (L.) Kuntze	Lamiaceae	fibrous root system	AM [55]	Dry rocky soils	He	S	Population-specific Zn tolerance [56,57]
*Dactylis glomerata* L.	Poaceae	Fibrous root system	AM [26]	Cultivated soils	He	CSR	Population-specific Zn tolerance [20]
*Dioscorea communis* (L.) Caddick & Wilkin	Dioscoreaceae	Rhizome	AM [25]	Calcareous, well-drained soils	L	CS	Not known
*Draba verna* L.	Brassicaceae	Fibrous root system	Not known	Sandy and calcareous soils	Th	CR	Not known
*Eryngium campestre* L.	Apiaceae	Rhizome	AM [26]	Sandy soils	Ge	SR	Population-specific Zn tolerance [16]
*Euphorbia cyparissias* L.	Euphorbiacee	Fibrous root system	AM [26,58]	Calcareous soils	He	S	Population-specific Zn tolerance [58]
*Festuca ovina* sl L.	Poaceae	Fibrous root system	AM [26,27]	Dry soils	He	CSR	Population-specific Zn tolerance [59]
*Galium aparine* L.	Rubiaceae	Slender root system	AM + NM [26]	Cultivated soils	Th	CR	Population-specific Zn tolerance [60]
*Galium corrudifolium* Vill.	Rubiaceae	Deep and fibrous root system	Not known	Dry soils	He	CR	Not known
*Helleborus foetidus* L.	Ranunculaceae	Rhizome	AM [26]	Well- drained, calcareous soil	Ge	CSR	Population-specific Zn tolerance [17]
*Hordeum murinum* L.	Poaceae	Fibrous root system	AM [26]	Dry and sandy soils, disturbed soils	Th	R	Population-specific Zn tolerance [61]
*Hornungia petraea* (L.) ex Rchb.	Brassicaceae	Fibrous root system	Not known	Sandy and rocky soils	Th	R	Not known
*Hypericum perforatum* L.	Hypericaceae	Rhizome	AM [26]	Roadside soils, dry soils	He	SR	Population-specific Zn tolerance [62]
*Juniperus oxycedrus* L.	Cupressaceae	Deep root system	AM [26]	Arid soils	Ph	CS	Population-specific Zn tolerance [63]
*Lactuca perennis* L.	Asteraceae	Deep root system	Not known	Rocky and calcareous soils	He	S	Not known
*Lepidium draba* L.	Brassicaceae	Rhizome	NM [64]	Roadside soils	Ge	CR	Population-specific Zn tolerance [65]
*Lysimachia arvensis* (L.) U. Manns & Anderb.	Primulaceae	Fibrous root system	AM [25]	Cultivated and sandy soils	Th	R	Not known
*Mibora minima* (L.) Desv.	Poaceae	Slender root	Not known	Sandy soils	Th	R	Population-specific Zn tolerance [66]
*Noccaea caerulescens* (J.Presl & C.Presl) F.K.Mey	Brassicaceae	Fibrous root system	AM [26]	Well-drained soils, cultivated soils	He	CS	Species-wide Zn tolerance [20], Zn/Cd/Ni hyperaccumulator [3]
*Pilosella officinarum* Vaill.	Asteraceae	Rhizome	AM [67]	Dry soils	He	CS	Population-specific Zn tolerance [68]
*Pinus sylvestris* L.	Pinaceae	Deep root system	EC [26]	Dry soils, sandy and rocky soils	Ph	CS	Population-specific Zn tolerance [69], Zn accumulator [69]
*Pistacia terebinthus* L.	Anacardiaceae	Deep root system	AM [26]	Calcareous soils	Ph	CS	Population-specific Zn tolerance [70]
*Plantago lanceolata* L.	Plantaginaceae	Fibrous root system	AM [25]	Neutral to calcareous soils, rangelands	He	CSR	Population-specific Zn tolerance [20], Zn- accumulator [62]
*Poa annua* L.	Poaceae	Fibrous root system	AM + NM [26]	Well-drained soil	Th	R	Population-specific Zn tolerance [71]
*Poterium sanguisorba* L.	Rosaceae	deep-root system	AM [72]	Roadside soils, Rocky soils	He	CS	Population-specific Zn tolerance [20]
*Pyrus spinosa* Forssk.	Rosaceae	deep-root system	AM + NM [26]	Dry soils, Rocky soils	Ph	C	Not known
*Quercus ilex* L.	Fagaceae	deep-root system	EC [26]	Calcareous soils, well-drained soils	Ph	CS	Population-specific Zn tolerance [18], Zn- accumulator [18]
*Quercus pubescens* Willd.	Fagaceae	deep-root system	EC [26]	Well-drained soils, Calcareous soils	Ph	C	Not known
*Ranunculus bulbosus* L.	Ranunculaceae	Tuberous root system	AM [26,27]	Calcareous soils, Stony soil	Ge	CSR	Population-specific Zn tolerance [21]
*Reseda lutea* L.	Resedaceae	deep-root system	AM + NM [26,27]	Sandy soils, roadside soils, rangelands	He	CSR	Population-specific Zn tolerance [20]
*Rosa canina* L.	Rosaceae	deep-root system	AM [26]	Poor soils, sandy soils	Ph	CS	Population-specific Zn tolerance [73]
*Rubia peregrina* L.	Rubiaceae	Rhizome	AM [26]	Dry soils, well-drained soils	He	CS	Population-specific Zn tolerance [19]
*Rubus ulmifolius* Schott	Rosaceae	Rhizome	AM [26]	Calcareous soils	Ph	CS	Population-specific Zn tolerance [19]
*Rumex intermedius* D.C.	Polygonaceae	deep-root system	AM [26]	Rocky soils, dry soils	He	CSR	Not known
*Ruscus aculeatus* L.	Asparagaceae	Rhizome	AM [25]	Dry soils, rocky soils	Ch	CS	Population-specific Zn tolerance [19]
*Scabiosa atropurpurea* L.	Caprifoliaceae	Deep root system	Not known	Sandy soils, rangelands	He	CSR	Population-specific Zn tolerance [74]
*Scrophularia lucida* L.	Scrophulariaceae	Slender roots	Not known	Dry calcareous soils	He	CSR	Population-specific Zn tolerance [75]
*Sedum acre* L.	Crassulaceae	Slender roots	AM [26]	Rocky soils	Ch	S	Population-specific Zn tolerance [36]
*Sedum annuum* L.	Crassulaceae	Slender roots	Not known	Dry soils	Th	R	Not known
*Senecio vulgaris* L.	Asteraceae	Deep root system	AM [26,76]	Cultivated soils	Th	R	Population-specific Zn tolerance [36]
*Silene vulgaris* (Moench) Garcke	Caryophyllaceae	Slender roots	NM [27]	Well-drained soils, rangelands	He	CSR	Population-specific Zn tolerance [8]
*Smilax aspera* L.	Smilacaceae	Rhizome	AM [25]	Dry soils	L	CS	Population-specific Zn tolerance [77]
*Thymus vulgaris* L.	Lamiaceae	Fibrous root system	AM [26]	Dry soils	Ch	CS	Population-specific Zn tolerance [20]
*Trifolium pratense* L.	Fabaceae	Deep root system	AM + NM [26,27]	Meadows, woods	He	CSR	Population-specific Zn tolerance [27]
*Ulex parviflorus* Pourr.	Fabaceae	Deep root system	AM [26]	Dry, rocky soils, poor soils	Ph	CS	Not known
